# Meeting reports

**DOI:** 10.1155/S1463924682000479

**Published:** 1982

**Authors:** F. L. Mitchell, John G. Williams


Journal of Automatic Chemistry, Volume 4, Number 4 (October–December 1982), pages 191–192

### Meeting reports

### International Congress on Automation in the Clinical Laboratory: Barcelona, 19–22 April 1982

The impact of automation on clinical chemistry has been increasingly felt in the speciality since the late 1950s, and in 1979 the Sociedad Española de Quimica Clinica organized a national congress on the subject in Barcelona. The Spanish society was sufficiently encouraged by the success of this meeting to organize a full congress in 1982, this time on an international scale, and expanded to cover other branches of pathology.

Dr Roman Galimany was responsible for the overall organization of both congresses and must be congratulated for his dedication and the successes achieved. The scientific programme of the last meeting was largely devised by the IFCC Expert Panel on Instrumentation, the intention being to cover as many aspects as possible of automation as currently understood in the three branches of pathology so far affected.

Professor Büttner set the scene by presenting a most illuminating address during the opening evening, entitled 'Technical evolution in the clinical laboratory'. He aptly quoted Sir Francis Bacon 'Neither the naked hand nor the understanding left to itself can effect much. It is by instruments and helps that work is done'.

The three plenary lectures on the morning of each day of the congress covered each speciality in turn (Professor Bonini, clinical chemistry; Professor Friedman, microbiology; and Dr Lewis, haematology). The remainder of the congress was taken up by seven symposia and two lectures by Professor Hjelm on nomenclature. The titles of the symposia were—Solid phase chemistry, Systems for kinetic measurements, Use of immobilized enzymes, Instruments for developing countries, Cost analysis, Computer interfaces and patient identification and New technology allowing pathology near the patient.

The Palacio de Congresos is one of the largest congress centres in Europe and comfortably held the 600 congress members together with a sizeable instrument exhibition.

The superb, historical city of Barcelona, blessed with excellent weather, provided accompanying members (and full members in their limited spare time) with a full social programme. Hospitality at official receptions was fully up to Spanish tradition. The Ballet de la Opéra de Paris was in town to perform *Giselle*, and as a break from very long days of science, a fleet of coaches on Wednesday afternoon visited Sitges and Tarragona, finishing with a Catalan dinner and fiesta. Most members did not make their beds much before 3 a.m., but even so, Dr Lewis had an excellent audience for his plenary lecture on 'Automation in haematology' at 9 a.m. the next day.

If there are to be more international congresses on automation I am sure that few who were present at the first would be unhappy with a return to Barcelona.

**F. L. Mitchell**

### Flow Analysis II: Lund, 18–21 June 1982

The four-day symposium was arranged by the Analytical Section of the Swedish Chemical Society. Flow Analysis I was held in Amsterdam three years ago and the symposium in Lund reaffirmed the world-wide interest in analyses performed within flowing stream reactors. About 150 participants attended the conference originating from 16 countries, including some as far away as China and Japan. The conference consisted of lecture sessions, posters and a social programme.

Not surprisingly, perhaps, most of the lectures focused on flow-injection analysis. Heavy weighting was applied to the overall content of the symposium from lectures centred on theoretical dynamic flow parameters. The lectures were a little lacking in analytical achievement; they were almost void of examples of chemical analysis where improvement was unmistakably derived from expositions of practice by theories of fluid mechanics. So much attention was given to non-segmented stream analysis that one could be easily deluded that almost all chemical analysis could be performed by adding one or two reagents to a flowing stream: the samples for such analysis having undergone extensive clean-up and preparation. The international struggle covertly claiming the renascence of unsegmented stream analysis seems at times devoted to creating personal recognition from an anti-genius. The real genius being the person who put the bubble in a flowing stream initially.

The symposium was divided into four lecture sessions; each session being commenced by a plenary lecture. The four plenary lecturers were Professor R. Tijsesen, Professor J. Ruijicka, Professor H. A. Mottola and Dr H. Poppe. Professor Tijsesen opened the lecture session with a plenary lecture principally describing axial and radial dispersion phenomena in flow analysis. Professor Ruijicka discussed recent developments in flow-injection analysis including gradient techniques and hydrodynamic injection. The use of enzymatic preparations in analytical continuous-flow systems was described by Professor Mottola, and the introduction to the final session consisted of Dr H. Poppe of the University of Amsterdam discussing the performance of some liquid-phase flow-through detectors.

There were many well-prepared and interesting lectures presented at the symposium in a friendly international atmosphere. Professor J. N. Miller described the use of flow-injection analysis in the development of luminescence immunoassays. Ample time was allocated to an excellent poster session. The conference was generally well organized with only minor 'hiccups'.

The charming city of Lund, founded in 1020 by King Canute, was a good choice of venue. Refreshments were supplied by Elsevier Scientific Publications Company and Tecator AB/Bifok-Optilab. The course dinner-dance was held in the splendid surrounds of the Grand Hotel in the city centre.

**John G. Williams**

### Erba Science 1982 Capillary Gas Chromatography Users' Forum

Erba Science (UK) Ltd, the British subsidiary of Carlo Erba Strumentazione of Milan, manufacturer of instruments for gas chromatography and other analytical procedures, held its annual Capillary Gas Chromatography Users' Forum at Nottingham University's Medical School on Wednesday, 7 July 1982. As in previous years, the day's programme of papers by guest speakers was combined with an exhibition by 10 companies manufacturing equipment for gas chromatography and related techniques.

Seven papers were presented, including an historical review of gas chromatography (GC): 'HRGC—how we reached where we are today' by Professor K. Grob. The audience was reminded that HRGC in glass capillary columns was an English invention by Professor Desty's group at British Petroleum in 1959, which was motivated by the wish to replace the costly steel capillaries then in use with glass as a readily available and cheap material. Professor Grob traced the history of the glass column through the early difficulties in establishing stable coatings, through the development of silica columns, to the present day. He drew attention to the need for analysts to move away from the tendency to judge GC on criteria arising from historical experience with packed columns and to evaluate its positive aspects in their own right.

E. Bailey of the MRC's Toxicology Unit read a paper on the 'Determination of alkylated amino acids in haemoglobin using HRGC/mass spectrometry'. He pointed out that the monitoring of environmental pollutants can put large demands on available analytical resources and then described his own method for determining low environmental concentrations of mutagens and carcinogens from human blood samples. After the isolation of globin, the addition of ether as a standard, and hydrolysis, the resulting hydrolysate is chromatographed. Quantitation is by capillary GC/MS with single or multiple ion-detection. It is shortly hoped to apply this method to screening industrial workers.

Computerized peak identification in capillary GC was discussed by J. A. Lomax of the Rowett Research Institute. He considered the need to examine the structure of polysaccharides in the cell walls of grass and other forage crops, and the need to be able to achieve this without the regular availability of a mass spectrometer. The linking of a GC with an available computer for peak identification has allowed this to be achieved. Of particular interest was his description of the work necessary to establish retention data stable enough to allow valid computer analysis.

Forensic applications of GC were outlined by the Metropolitan Police Forensic Science Laboratory's Dr L. W. Russell. He described the use of headspace analysis for detecting the presence of petroleum products at the scene of arson from the small quantities that tended to remain in the resulting debris. Dr Russell described a method involving a pre-concentration step where a measured volume of headspace is drawn through a trap containing a porous polymer Tenax-GC. The trap—a small glass tube—with its contents is then inserted into the GC's injection port. A considerable advantage in this application is the much larger volume of headspace available for analysis.

Erba Science's Graham Wardall presented a paper on the 'Optimization of on-column injection with respect to solvent choice and oven temperature'. Mr Wardall outlined developments in chromatography since the introduction of the on-column injector in 1978. He reminded his audience that difficulties of poor peak shape could be traced to factors arising from incorrect injection temperature and column flooding, and outlined the developments that now allow higher temperatures to be applied and so improved results obtained.

R. J. Law from MAFF's Directorate of Fisheries Research Laboratory discussed the use of GC/MS as a tool for environmental studies. His laboratory uses GC and GC/MS extensively for the characterization of mixtures of organic compounds present in samples of water, fish tissue, sediments and industrial effluents. Amongst topics covered were the use of multiple ion detection for quantitative analysis for organometallic compounds in organic samples; examples included the detection of traces from marine anti-fouling paints in shellfish and measuring levels of alkyl lead discharged by petrol-additive manufacturers into canal-water.

Sets of bound Users' Forum papers are available from Erba Science. The company are now planning their 1983 meeting and would welcome offers of papers and enquiries from potential exhibitors.

*Further information from Erba Science Ltd, Headlands Trading Estate, Swindon, Wiltshire SN2 7JQ, UK. Tel.: 0793 33551.*

### SYMPOSIUM ANNOUNCEMENT

*The selection and use of liquid/solid separation equipment*

On 24 November the Institute of Chemical Engineers, in collaboration with the Filtration Society, are holding a one-day symposium on liquid/solid separation at UMIST, Manchester, UK. The emphasis throughout the day will be on the practical aspects of the subject. Pressure, vacuum and centrifugal filters will be featured, as well as the latest developments in filter media. The adjoining exhibition will allow symposium delegates to see the equipment featured in the papers presented and to discuss their problems with manufacturers. The symposium will start at 9·00 a.m. and finish at about 5·45 p.m. The cost (including lunch and reprints) is £35·00 for members of the I.Chem.E. or the Filtration Society and £40·00 for non-members.

*Further details of the symposium and exhibition can be obtained from Mr A. L. Clarke, Diamond Shamrock (UK) Ltd, PO Box 1, Eccles, Manchester M30 0BH, UK. Tel. 061 789 7300.*

